# Evaluation of microRNA-223 and microRNA-125a expression association with STAT3 and Bcl2 genes in blood leukocytes of CLL patients: a case–control study

**DOI:** 10.1186/s13104-020-05428-0

**Published:** 2021-01-11

**Authors:** Nader Davari, Fatemeh Ahmadpour, Ali Asghar Kiani, Mozhgan Azadpour, Zari Tahannejad Asadi

**Affiliations:** 1 Thalassemia & Hemoglobinopathy Research Center, Health Research Institute, Ahvaz Jundishapur University of Medical Sciences, Ahvaz, Iran; 2 Department of Clinical Biochemistry, Faculty of Medicine, Ahvaz Jundishapur University of Medical Sciences, Ahvaz, Iran; 3 Department of Hematology and Blood Transfusion, Lorestan University, Khoramabad, Iran; 4 Hepatitis Research Center, Lorestan University of Medical Sciences, Khorramabad, Iran; 5 Razi Herbal Medicines Research Center, Lorestan University of Medical Sciences, Khorramabad, Iran; 6 Department of Laboratory Sciences, Faculty of Paramedicine, Ahvaz Jundishapur University of Medical Sciences, Ahvaz, Iran

**Keywords:** Chronic lymphocytic leukemia, MicroRNA-223, MicroRNA-125a, BCL2, STAT3, Prognostic

## Abstract

**Objective:**

In chronic lymphocytic leukemia (CLL), lack of expression or dysregulation of some special miRs disrupts apoptosis of malignant cells; thereby miR expression can enhance cell proliferation, disease progression and decrease patient survival.

**Results:**

30 CLL patients and 20 healthy individuals participated in the study. RNA was extracted to evaluate the expression of miR-125, miR-223, BCL-2 and signal transducer and transcription 3 activator (STAT3) genes; quantitative Real Time- PCR (Q-RT-PCR) was performed. MiR-125a and miR-223 expression decreased in the patients compared to the control group (P-Value:0.001). BCL-2 and STAT3 which are the target genes of these two miRs, showed increased expression, in the patients compared to the control subjects (P-Value: 0.001 and P-Value: 0.64 respectively). A significant reverse relationship was found between miR-125a and BCl-2 expression and WBC count. Significantly, miR-223 expression was associated with smoking in patients (P-Value: 0.007). Also, these miRs may have regulatory effects by controlling white blood cell (WBC) production based on the inverse correlation with WBC count and hemoglobin (Hb) concentration. Finally, miR-223 can be used as a prognostic factor in CLL patients; miR-125a may be useful for evaluating the therapeutic approaches based on the inverse link with BCl-2.

## Introduction

Chronic lymphocytic leukemia (CLL) is a type of malignancy which is characterized by accumulation of lymphocytes (mostly B lymphocytes) in bone marrow (BM), lymph nodes, and peripheral blood (PB) [1]. In fact, it is a development and maturation disorder of lymphocytes. CLL is the most common adult malignancy in the western societies [2]; the average age of patients is 65 years and the incidence is twice as high in men [2, 3]. The main etiology of CLL has not been identified so far, and many factors and pathways had been investigated to describe the CLL pathogenesis [4].

Recent studies have shown that MicroRNAs (miRs) can be used as therapeutic tools for CLL patients; they play regulatory roles in gene transcription and expression [5, 6]. In fact, miRs are series of non-coding RNAs, consist of 20–22 nucleotides; the two major classes of target genes of miRs are tumor suppressors and oncogenes. In CLL, usually miRs which target tumor suppressor genes are impaired, while the function of miRs that target oncogenes is increased [7, 8]. MiR-125a has been recognized as a tumor suppressor in many cancers; it is effective in malignancies pathogenesis such as breast cancer by affecting many transcription factors such as STAT3 and ERBB2 [9, 10]. MiR-223 is another tumor suppressor which targets the STAT3 factor, similar to miR-125 [11, 12].

In this study, we aimed to evaluate miR-125a and miR-223 expression in CLL and control subjects; in the following we analyzed miRs expression relationship with the STAT3 and BCL2 genes expression, as well as their clinical symptoms.

## Main text

### Materials and methods

Study design: CLL patients were diagnosed based on BM aspiration, cytogenetic study, immunophenotype, and morphological findings. The patients (n = 30) under study were selected among those who referred to Ahvaz Baqaei hospital during 2018–2019. Inclusion criteria included CLL patients who had complete and accessible clinical information, without any underlying diseases; the malignancy was confirmed by two expert oncologists. Patients with underlying disease and who did not have complete clinical documents were excluded from the research. Also, 20 age matched control personals were entered the study.

10 ml of PB was collected from each individual in EDTA anticoagulant tubes. All demographic data of studied individuals are listed in Table1. 60% of CLL patients had agricultural related occupations, 20% were employees and 16.7% were housewives; 3.3% of them were freelancers.

**Table 1:** Demographical information of patients and controls

Variables	Mean ± SD		P-value
	Patient (n = 30)	Control (n = 20)	
Age(year)	63.23 ± 6.35	54.93 ± 5.53	0.51
Sex(Female/Male)	11/19	10/10	0.26
Height	168.86 ± 17.17	169.93 ± 8.75	0.67
Weight	78.64 ± 33	73.18 ± 17.88	0.90
BMI (kg/m^2^)	28.54 ± 14.23	25.08 ± 4.39	0.87
Hb (g/dl)	10.46 ± 1.91	14.10 ± 1.01	0.08
WBC (10^3^/mm^3^)	52.28 ± 66.99	7.06 ± 1.18	< 0.0001
PLT (10^3^/mm^3^)	105.76 ± 57.61	224.31 ± 47.99	< 0.0001

This study was approved by the local ethics committee of Ahvaz Jundishapur University of Medical Sciences (AJUMS.REC.1397.763); the informed consent was completed by whole of subjects.

### miRNA expression analysis

After sample collection, PB mononuclear cells were isolated by Ficoll-Hypaque density gradient centrifugation method; mRNA isolation was performed according to the Biomaxell kit manufacturer protocol. The extracted RNA was evaluated by light absorption at 260 nm; in the next step cDNA synthesis was performed according to the yektatajhiz azma kit protocol. cDNA synthesis temperature cycle conditions have been described as following, initial denaturation for 5 min at 70℃, 1 h at 42℃, and finally 5 min at 70 ℃.

List of the used materials for cDNA synthesis is as follows, 1000 ng total RNA, 1 μL Oligo dt primer, 4 μL first strand buffer, 1 μL dNTP, 1 μL M-Mlv, 0.5 μL Rnase inhibitor and 20 μL Diethyl pyrocarbonate (DEPC) water.

Dedicated primers were used for stem loop miR-125a, stem loop miR-223 and stem loop U6, instead of oligo dt primer for cDNA synthesis of microRNAs. The cited stem loops sequences are as follows.

Stem Loop miR-125a-5P: 5′-GTCGTATCCAGTGCAGGGTCCGAGGTATTCGCACTGGATACGACtcacag-3′.

Stem Loop miR-223-3P: 5′-GTCGTATCCAGTGCAGGGTCCGAGGTATTCGCACTGGATACGACtggggt-3′.

Stem Loop U6: 5′-GTCGTATCCAGTGCAGGGTCCGAGGTATTCGCACTGGA TACGACaaaata-3′.

The temperature cycle for cDNA synthesis included initial denaturation for 5 min at 70℃ and 1 h at 37℃, and 5 min at 70 ℃.

Then, Q-RT-PCR was used to evaluate the expression of miR-125, miR-223, BCL-2 and STAT3. Temperature cycle included initial denaturation for 15 min at 95℃,)15 s at 95℃ and 1 min at 60℃ (with 40 cycles in 20 μL of PCR master mix. It contained 10 μL of SYBR-Green QPCR Master Mix (amplicon), o.4 μL forward primer, 0.4 μL reverse primer, 1 μL cDNA and 8.2 μL of RNase free water for GAPDH, BCL-2 and STAT3; in addition, the following materials were added for U6, miR-125 and miR-223, 10 μL of SYBR-Green QPCR Master Mix, o.8 μL forward primer, 0.8 μL reverse primer, 1 μL cDNA and 7.4 μL of RNase free water.

U6 and GAPDH were used as internal controls. mRNA primers were designed by using human Nucleotide BLAST program and based Stem Loop assay for miRs [13]. The sequences of the used primers are listed in Table 2.

**Table 2:** Sequence primers used for Q-RT-PCR

	Forward	Reverse
miR-125	5′-CGGCGCTCCCTGAGACCCTTTA-3′	5′-GTGCAGGGTCCGAGGT-3′
miR-223	5′-GCAGAGTGTCAGTTTGTCAAAT-3′	5′-GTGCAGGGTCCGAGGT-3′
U6	5′-CTCGCTTCGGCAGCACA-3′	5′-GTGCAGGGTCCGAGGT-3′
BCL2	5′-AGGATAACGGAGGCTGGGATG-3′	5′-CATATTTGTTTGGGGCATGT-3′
STAT3	5′-GCTGCCCCATACCTGAAGAC-3′	5′-GGTGAGGGACTCAAACTGCC-3′
GAPDH	5′-GGTCGGAGTCAACGGATTTGG-3′	5′-TGATGACAAGCTTCCCGTTCT-3′

### Statistical analysis

Statistical analyses were performed using SPSS 23.0. The data were expressed as mean ± SD; P values < 0.05 were considered statistically significant. Gene expression was analyzed by 2^− ΔΔCT^ method. Mann–Whitney, Roc curve and spearman tests were applied for analysis of the data.

## Result

### Expression of miR-125a, miR-223, BCL2 and STAT3

**Fig. 1:**
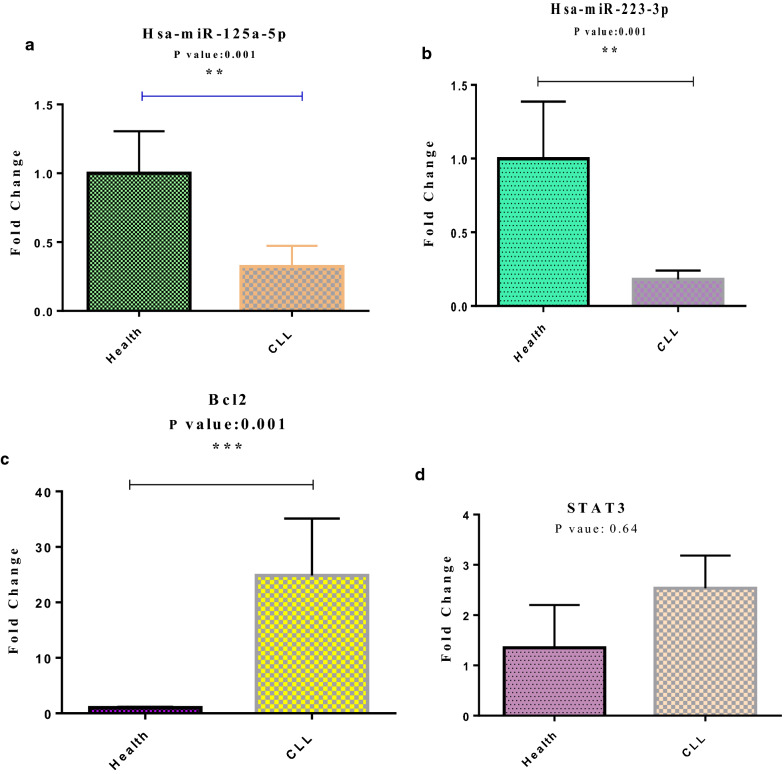
Expression of miR125a **a**, miR-223 **b**, BCL-2 **c** and STAT3 **d** in healthy and patient groups

miR-125a and miR-223 expression was increased in healthy individuals compared to the CLL patients, which was statistically significant (P-Value: 0.001). The analysis indicated that STAT3 and BCL2 expression was higher in the CLL patients. There is insufficient evidence to support the claim that STAT3 expression differs meaningfully between the two groups (P-Value: 0.64), while BCL2 expression significantly vary between the case and control group (P-Value: 0.001) (Fig. 1).

### Association between miR-125a and miR-223 with demographical information in patients

Based on Additional file 1: Table S1, patients who smoked (n = 5) significantly express lower level of miR-223 (P-Value: 0.001) compared to non-smokers (n = 25). However, there was no significant relationships between other parameters and the cited miRs expression.

### Correlation between miR-125a and miR-223 with laboratory parameters

According to the results, the expression level of miR-125a in healthy group was correlated with BCL2 gene, inversely (P-Value: 0.02 and total population (case and control) P-value: 0.001). On the other hand, there was a significant inverse relationship between white blood cell count (P-Value: 0.01) in the patients group. A significant direct correlation was found between miR-125a expression and hemoglobin concentration in the patient and control individuals (P value: 0.003). (Additional file 1: Figure S2). Also, the expression level of miR-223 was inversely correlated with age in the patients group and the whole population (P value: 0.014, P-Value < 0.001 respectively).

In the whole population, a significant direct relationship was found between miR-223 expression and hemoglobin concentration (P value: 0.012); similar relationship was found between the two miRs expression (P value: 0.001). A significant inverse link was observed between miR-223 and white blood cell count (P value 0.002) (Additional file 1: Figure S1).

### ROC curve analysis

An effective and well-known method for evaluating a laboratory test (generally a diagnostic procedure) is receiver operating characteristic (ROC); the ROC curve can answer the question of whether a variable can be a suitable biomarker for a disease. This curve is based on sensitivity and specificity; the area under the curve indicates the diagnostic power of the test. According to the Additional file 1: Figures S3 and S4, miR-125a-5p, miR-223-3P and BCL2 gene expression are considered as useful biomarkers for CLL progression monitoring (p value < 0.001(.

## Discussion

CLL is one of the hematologic malignancy that characterized by accumulation of B-cells in PB and BM [14]. Due to the prevalence of CLL in Western societies, many studies have been done to identify therapeutic and prognostic markers [15, 16]. MiRs have wide variety of roles in cell structure and metabolism, but they appear to be most important in regulating hematopoiesis and producing different blood cell lines [17, 18]; miRs expression or function impairment is associated with some diseases, including CLL. The expression of some miRs can be associated with malignant cell proliferation inhibition or increased survival of patients [19, 20]. Recent studies have shown that some miRs can be used as a prognostic factor, since they target many involved genes in cell cycle and apoptosis [21].

Ahmadvand study evaluated miR-125a as a biomarker in CLL patients; decreased miR-125 expression was reported in the patients [22]. Another study by Rigolin and associates was performed to determine the association between miR-125a expression and genetic abnormalities in CLL patients; they discovered that reduced miR-125a expression is associated with more genetic abnormalities in CLL individuals [23]. As it was mentioned in the previous part, miR-125a expressed in lower level in the patients under the present study compared to the controls; the reduction was statistically significant (P-Value: 0.001) (Fig. 1a).

A study by Vicente.et al. [24] found that miR-223 expression was reduced in CLL patients lacking the immunoglobulin heavy chain mutation, which was associated with poor prognosis and disease progression. Another study by Zhou confirmed Vicenta’s findings; Zhou and coworkers declared that reduced miR-223 expression in CLL patients is associated with aggressive disease and decreased response to treatment [25]. In the present study, miR-223 reduced expression was observed in CLL patients, similar to miR-125a (P-Value: 0.001). It seems that miR-223 is a suitable marker to differentiate between the two groups of cancer and healthy individuals, based on the ROC Curve test for high percent, almost > 80% (Fig. 1b and Additional file 1: Figures S4).

Given that, miR-125a and miR-223 are involved in the pathogenesis and treatment response of patients by targeting some genes; evaluating their association with clinical and laboratory parameters may provide useful evidences to improve the importance of these miRs as a prognostic factor. The study by Stamatopoulos.et al. [11] reported a significant relationship between miR-223 and Binet staging system of CLL patients (P-Value: 0.01); however, there was no significant relationships between miR-223 and gender (P-Value: 0.49). Toward relation of demographic information and clinical findings of CLL patients, Zhou.et al. did not observe any significant relationships between miR-223, age and leukocyte count (P- Value: 0.23 for age and P-Value: 0.21 for leukocytes).

In contrast to age parameter, miR-223 expression was meaningfully related to the patients stage based on the Binet system (P-Value: 0.00) [25]. In the present study none of the cited miRs expression was correlated to the demographic data, except miR-223 which was significantly associated with smoking in CLL patients (P-Value: 0.007) (Additional file 1: Table S1). In fact, these two miRs serve as tumor suppressors which increase therapy response and patient survival; they prevent malignant cells production and induce apoptosis.

Thus, it has been proved that the two BCL-2 and STAT3 genes, which are involved in the proliferation and inhibition of malignant cell apoptosis, are targeted by these two miRs. To this end, Tong.et al. [26] designed a study to investigate the effect of miR-125a on BCL-2 expression in colon cancer cells. They concluded that increased expression of miR-125a is associated with decreased expression of BCL-2, in colon cancer cells. A new finding in our clinical study is invers relation of miR-125a expression to bcl2. It suggests the important role of miR-125a to prevent CLL progression by BCL-2 expression reduction. It could be a therapeutic goal in the future (Additional file 1: Figure S2).

Bozec and associates reported reverse correlation expression of miR-223 and STAT3 in cancer cells; STAT3 is one of the key signaling factors in malignant cell proliferation [27]. According to the results of the present study, BCL-2 and STAT3 expression was higher in the patients than the healthy group; this difference was statistically significant for the BCL-2 as opposed to the STAT3 (P-Value: 0.001 for BCL-2 and P-Value: 0.64 for STAT3) (Fig. 1c, d).

## Conclusion

Finally, given that miR-125a and miR-223 increase in CLL patients and target both BCL-2 and STAT3, it can be concluded that miR-223 expression may be used as a suitable prognostic factor for CLL; miR125a is an appropriate marker for treatment based on the BCL2 link, too.

According to the inverse correlation of the two miRs with WBCs count and Hb concentration, it can be concluded that these miRs expression may have regulatory effect by controlling WBC proliferation. A significant inverse relationship between miR-223 and smoking suggests a new idea for further researches on smoking, miRs and related molecular signaling.

## Limitations

It is better to monitor the expression of miR-125 and miR-223 during the treatment procedure and evaluate the response to treatment. Evaluation of miR-125 and miR-223 expression in relation to patient survival has not been studied, yet; also, the relationship between these two and expressed CD markers, should be investigated, before and after treatment.

## Supplementary Information

**Additional file 1: Table S1.** Association between miR125a and 223 with clinico-pathological parameters. **Figure S1.** Inverse and significant relationship between miR-125a gene expression and white blood cell count in patients. Spearman correlation, p value ≤0.05. **Figure S2.** Significant inverse relationship between miR-125a ΔCT and BCL2 ΔCT in healthy group. Spearman correlation, p value ≤0.01. **Figure S3.** The area under the curve corresponding to the ROC analysis of Has-miR-125a and Has-miR-223 variables in two patient and healthy groups. **Figure S4.** The area under the curve corresponding to the ROC analysis of BCL2 in two healthy and patient groups.

## Data Availability

The datasets used analyzed during the current study are available from the corresponding author on reasonable request.
