# Supplementary material for: Evaluation of microRNA-223 and microRNA-125a expression association with STAT3 and Bcl2 genes in blood leukocytes of CLL patients: a case–control study
Source: BMC Res Notes. 2021 Jan 11;14:21. doi: 10.1186/s13104-020-05428-0 (PMC8095339; doi:10.1186/s13104-020-05428-0)
Supplement: Supplementary file 1 — Additional file 1: Table S1. Association between miR125a and 223 with clinico-pathological parameters. Figure S1. Inverse and significant relationship between miR-125a gene expression and white blood cell count in patients. Spearman correlation, p value ≤0.05. Figure S2. Significant inverse relationship between miR-125a ΔCT and BCL2 ΔCT in healthy group. Spearman correlation, p value ≤0.01. Figure S3. The area under the curve corresponding to the ROC analysis of Has-miR-125a and Has-miR-223 variables in two patient and healthy groups. Figure S4. The area under the curve corresponding to the ROC analysis of BCL2 in two healthy and patient groups. [file 13104_2020_5428_MOESM1_ESM.docx]

Table. S1. Association between miR125a and 223 with clinico-pathological parameters.

| P-Value | | Variables |
| --- | --- | --- |
| miR-223 | miR-125a |  |
| 0.23 | 0.56 | BINET Stage |
| 0.55 | 0.72 | RAY Stage |
| 0.52 | 0.90 | Organomegaly |
| 0.85 | 0.73 | Sex |
| 0.007 | 0.44 | Smoking |
| 0.46 | 0.15 | History of family disease |
| 0.14 | 0.99 | Recurrence disease |
| 0.58 | 0.42 | Job |

Figure.S1. Inverse and significant relationship between miR-125a gene expression and white blood cell count in patients. Spearman correlation, p value ≤0.05.

Figure.S2. Significant inverse relationship between miR-125a ΔCT and BCL2 ΔCT in healthy group. Spearman correlation, p value ≤0.01


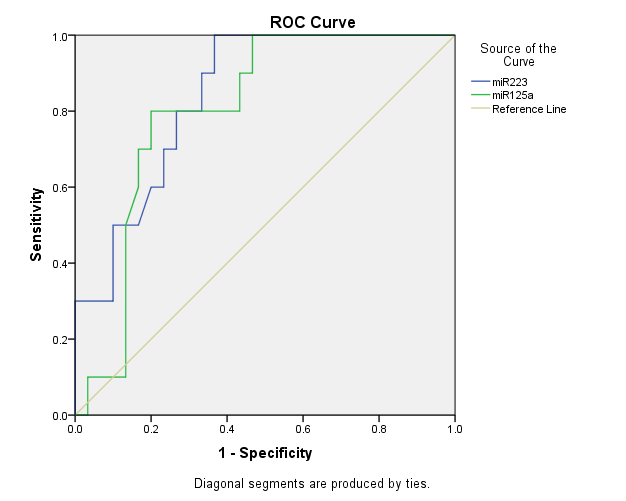
 Figure.S3. The area under the curve corresponding to the ROC analysis of Has-miR-125a and Has-miR-223 variables in two patient and healthy groups.


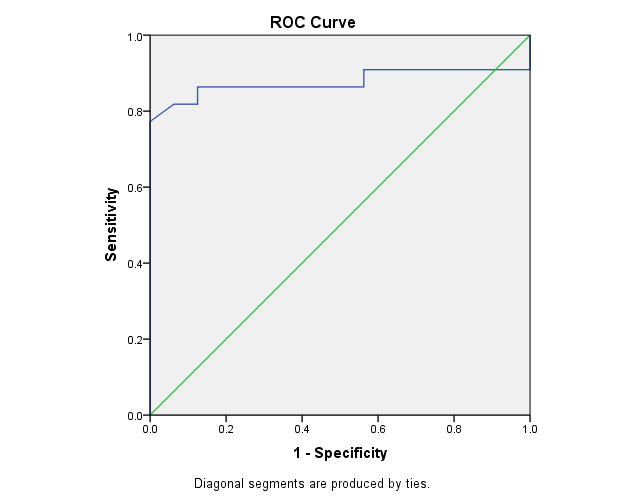
 Figure.S4. The area under the curve corresponding to the ROC analysis of BCL2 in two healthy and patient groups.
